# A Three-Dimensional-reconstruction-based study on the ocular volume of Chinese children with high myopia

**DOI:** 10.1186/s12886-021-02078-z

**Published:** 2021-09-07

**Authors:** Xiaodan Jiang, Hongwei Deng, Chun Lung, Fanyin Wang, Shuang Li, Yanni Jiang, Mingyue Wang

**Affiliations:** 1 Ophthalmology Department, Shenzhen Shekou People’s Hospital, No.36 Gongye 7th Road, Shekou, Nanshan District, 518067 Shenzhen, China; 2 Department of Strabismus & Pediatric Ophthalmology, Shenzhen Eye Hospital affiliated to Jinan University, The School of Optometry of Shenzhen University, No.18 of Zetian Street, Futian District, 518000 Shenzhen, China; 3 CAS-Medi-Vision Company Limited, Zhuhai, China

**Keywords:** Children, High myopia, Ocular volume, Computed tomography

## Abstract

**Background:**

Highly myopic eyes differ in morphology from emmetropic eyes, and the correct estimation of the vitreous volume is difficult. To explore an effective method to estimate ocular volume using refractive factors in children.

**Methods:**

This is a retrospective study of children with high myopia who visited the Shenzhen Shekou People’s Hospital (July-December 2018) before undergoing posterior scleral reinforcement surgery. Data on refractive factors and ocular 3D reconstruction imaging based on high-end CT were collected for linear correlation and linear regression analyses.

**Results:**

Ten patients (20 eyes) were included. There are nine males and one female. They were 4 to 12 years of age. The spherical equivalent ranges from + 0.25 to -20.00 D. The cylindrical equivalent ranges from − 0.50 to -6.25 D. The AL(axial length, AL) ranges from 21.78 to 33.90 mm. The corneal curvature (mean) ranges from 42.44 to 46.75. The 3D reconstruction of the CT images shows that the ocular volume ranges from 4.591 to 10.988 ml. The ocular volume of the 20 eyes decreases with the increase of diopter and total curvature, both presenting a linear trend, with the Pearson correlation coefficients being − 0.776 (*P* < 0.001) and − 0.633 (*P* = 0.003), respectively. The ocular volume of the 20 eyes increases with the increasing AL, also presenting a linear trend, with the Pearson correlation coefficient being 0.939 (*P* < 0.001).

**Conclusions:**

In children, the ocular volume is negatively and linearly correlated with the diopter and curvature, and positively and linearly correlated with the AL.

## Background

The incidence of high myopia is rising sharply worldwide. About 163 million people around the world have high myopia, accounting for 27 % of the total population [1–4]. Asia, especially East Asia, is a high incidence region of high myopia, and China is one of the countries with the highest incidence [4, 5]. High myopia can lead to myopia traction maculopathy, myopic choroidal neovascularization, and arched macular degeneration [6–8], and is, therefore, the second leading cause of low vision and blindness in the Chinese population [9].

There is a lack of effective ways to prevent or control high myopia. Existing treatments are focusing on controlling the increase of the axial length and curing common complications [10, 11]. Among them, surgical treatments include vitrectomy, internal limiting membrane removal, internal limiting membrane packing, posterior scleral reinforcement, and intravitreal injection of anti-vascular endothelial growth factor [12–14]. For example, for rhegmatogenous retinal detachment, one of the common complications of high myopia, intravitreal injection of exogenous filling is required in the reduction surgery for retinal detachment and posterior vitrectomy [15, 16].The amount of filling and intraocular pressure (IOP) control directly determines the prognosis of retinal reattachment [15, 16], but it is subject to subjective judgments as the vitreous volume of highly myopic eyes varies greatly between individuals [17]. Although some scholars have paid attention to the morphology of posterior scleral staphyloma and the thinning of intraocular optic nerve after the axial length elongation [18, 19], few scholars have studied the problem of enlarged eyeball volume, which is really a problem that needs the attention of surgeons before vitrectomy and posterior scleral reinforcement. In addition, the evaluation of the filling amount of silicone oil and the size of the customized artificial vitreous body requires the acknowledgment of the vitreous volume. A method to estimate the volume by diopter might provide quantitative data for clinical practice.

The in vivo measurements on the diameter line and eyeball shape for studies on ocular volume are rare in China. Using X-ray technology, Vohra et al. [20] reported in 2000 that the increase in the length of myopic eyes is much higher than that of width and height. Highly myopic eyeballs have an asymmetric growth, which is mainly reflected in the changes of the vitreous body [21]. Feng et al. [22] found that the total intraocular volume and vitreous volume of highly myopic eyes differ from those of emmetropic eyes, which is positively correlated with the diopter, while the volume of the anterior ocular segment and the crystalline lens of highly myopic eyes differ little from emmetropic eyes. Therefore, this evidence suggests that the increase of vitreous volume in highly myopic eyes leads to the increase of total intraocular volume, while the volume of the anterior ocular segment and the crystalline lens remains unchanged.

Nevertheless, data about vitreous volume in highly myopic eyes is missing. Therefore, this study aims to explore an effective method to estimate ocular volume using refractive factors. This could provide a simple method to estimate the vitreous volume without the need for sophisticated equipment and should provide data for individualized precision treatment for patients undergoing vitreoretinal surgery or needing implantation of an artificial vitreous body.

## Methods

### Study design and patients

This is a retrospective study of children with high myopia who visited the Ophthalmology Department of Shenzhen Shekou People’s Hospital from July to December 2018. The study was approved by the Medical Ethics Committee of Shenzhen Shekou People’s Hospital. The requirement for individual consent was waived by the committee because of the retrospective nature of the study.

The inclusion criteria are 1) < 18 years of age, 2) diagnosed with high myopia (>-6.0 D) of one or both eyes, and 3) with complete imaging information. The exclusion criteria are (1) a history of eye injury or (2) periorbital or paranasal sinus disease. The patients underwent ocular and periocular structure examinations using a high-end CT scanning system because they needed to be ruled out orbital deformity and paranasal sinus inflammation before undergoing monocular post-capsule reinforcement surgery.

### Data collection

Detailed information on the ocular refractive factors of the included patients was collected. Sex, age, vision, corneal curvature, ocular AL, and refraction information were recorded. After pupil dilation with one drop of compound tropicamide eye drops 0.5 % (Shenyang Sinqi Pharmaceutical Co., Ltd., Shenyang, China), the patients underwent optometry and ocular bio-measurements, with relevant data recorded. An RT 5100 integrated optometrist system (NIDEK, Tokyo, Japan) was used for computerized optometry, and a LS 900 biometer (Haag-Streit Holding, Koniz, Switzerland) was used for optical biometry. In the ametropia examination, each subject underwent five repeated binocular refraction examinations, and a mean value was calculated from the five measured values recorded.

### Computed Tomography scan and Three-Dimensional reconstruction

Three-Dimensional Computed Tomography Scan Images were acquired using a 256-slice multiple detector computed tomography (Revolution CT) scanner (GE Medical Systems LLC, Waukesha, WL 53,188, USA), with 256 × 0.625mm collimation. No contrast agent was given. Reconstrutions were obtained at using a smooth reconstruction kernel, with a slice thickness of 0.625 mm and increment of 0.3125. Revolution CT datasets were analyzed using the 3D Medtech Reconstruction System by CAS-Medi-vision Company Limited.

**Fig. 1:**
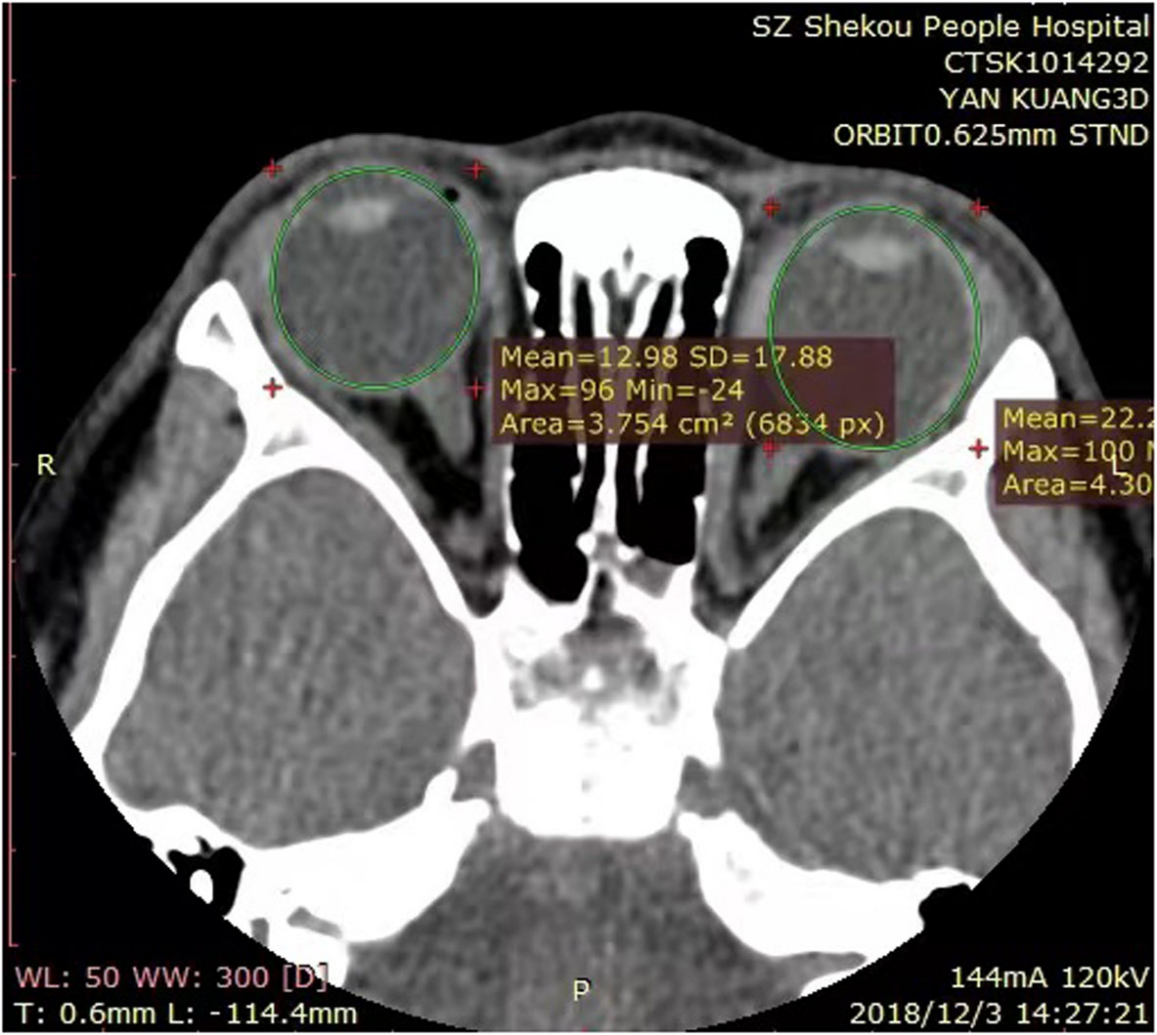
Pearson correlation analyses between the ocular volume and **A** diopter (*r*=-0.776, *P* < 0.001), **B** corneal curvature (*r*=-0.633, *P* = 0.003), and **C** axial length (*r* = 0.939, *P* < 0.001) in 20 eyes from 10 children

Figure 1 3D-CT screenshots for the patient with myopia 3 diopters in right eye and 14.5 diopters in left eye are two different eyeballs in size shows how we get the original plane data. The first step: A slice of CT images. The second step: Draw lines on the inner surface of the eyeball (shows in green line in Fig. 1). The third step: reconstruction of the 3CT.

### Statistical method

SPSS 20.0 (IBM Corp., Armonk, NY, USA) was used for statistical analysis. Continuous data conforming to the normal distribution are expressed as means standard deviations(±); those not conforming to the normal distribution are expressed as medians (ranges). Categorical data are expressed as 5 (%). Pearson correlation and regression analyses were performed. Two-sided *P*-values < 0.05 are considered statistically significant.

## Results

### Characteristics of the patients

Ten patients (20 eyes) were included. There are nine males and one female. The age range from3 to 12, mean 6 years of age(6 ± 2.90). The spherical equivalent ranges from + 0.25 to -20.00 D. The cylindrical equivalent ranges from − 0.50 to -6.25 D. The axial length ranges from 21.78 to 33.90 mm. The corneal curvature (mean) ranges from 42.44D to 46.75D. The 3D reconstruction of the CT images shows that the ocular volume ranges from 4.591 to 10.988 ml.

### Correlation analyses

**Fig. 2:**
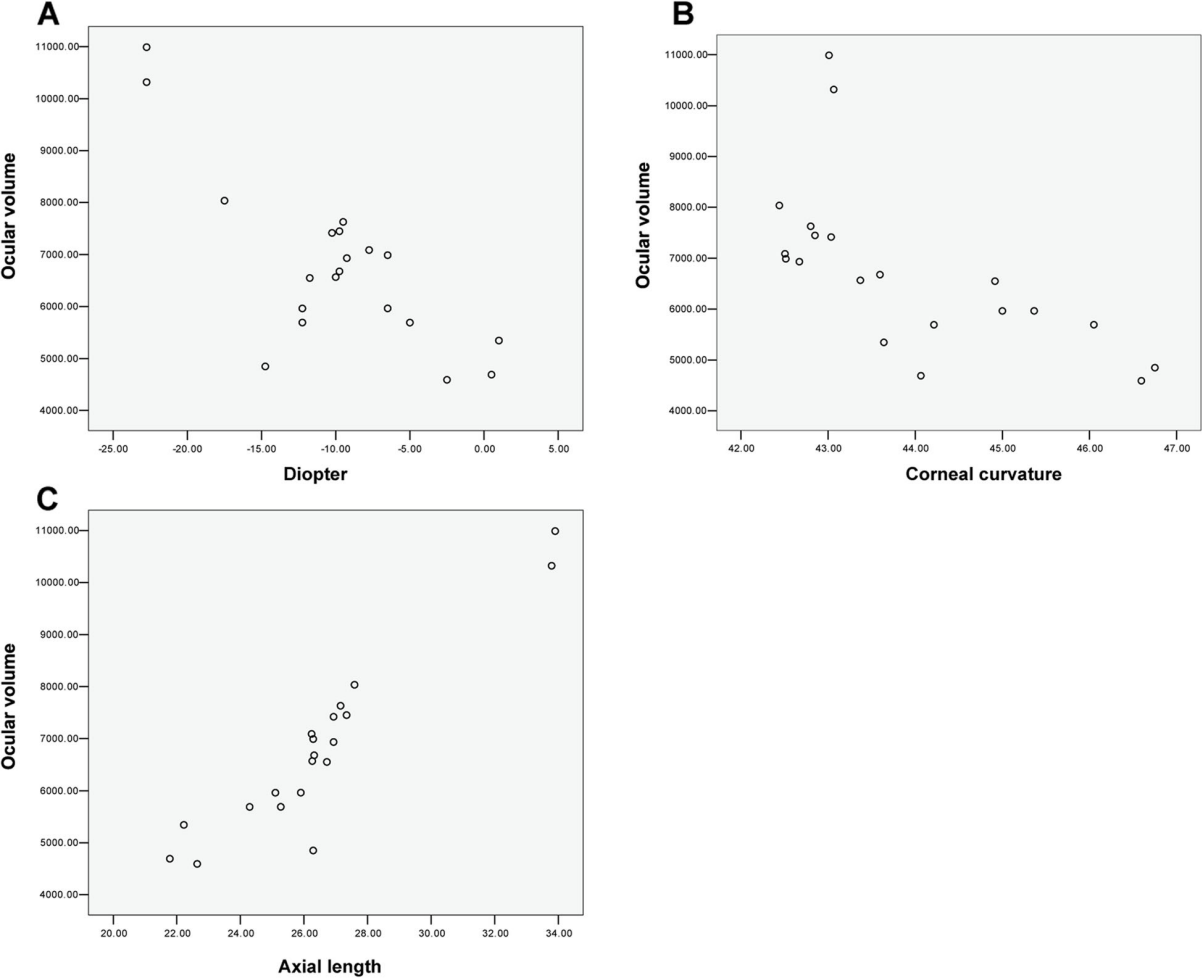
CT screenshots for the patient with myopia 3 diopters in right eye and 14.5 diopters in left eye

As shown in the scatter diagram in Fig. 2, the ocular volume of the 20 eyes decreases with the increase of diopter and total curvature, both presenting a linear trend, with the Pearson correlation coefficients being − 0.776 (*P* < 0.001) and − 0.633 (*P* = 0.003), respectively. The ocular volume of the 20 eyes increases with the increasing AL, also presenting a linear trend, with the Pearson correlation coefficient being 0.939 (*P* < 0.001). Hence, the ocular volume is negatively and linearly correlated with the diopter and curvature, and positively and linearly correlated with the AL.

### Linear regression analyses

The linear regression equation between ocular volume and total diopter was y = 4741.596-203.714x. In this case, the determination coefficient was 0.603, indicating that the total diopter accounted for only 60.3 % of the volumetric change. The linear regression equation between ocular volume and curvature was y = 39,851-753.144x. In this case, the determination coefficient was 0.401, indicating that the curvature accounted for only 40.1 % of the volumetric change. The linear regression equation between ocular volume and AL was y = 6814.273 + 513.673x. In this case, the determination coefficient was 0.939, indicating that the AL accounted for 93.9 % of the volumetric change.

## Discussion

Highly myopic eyes differ in morphology from emmetropic eyes, and the correct estimation of the vitreous volume is difficult [17]. This study aims to explore an effective method to estimate ocular volume using refractive factors in children. Using high-end CT 3D reconstruction data, the results indicate that in children, the ocular volume is negatively and linearly correlated with the diopter and curvature, and positively and linearly correlated with the AL.

Highly myopic eyeballs have asymmetric growth, as characterized by the elongation of the ocular equator, expansion of the posterior pole, and multi-directional and spherical ocular expansion [21]. Atchison et al. [23] showed that compared with emmetropic eyes, myopic eyes are elongated, more in length than in height, and even less in width. Bron et al. [24] suggest that the bone wall around the eyeball is closer to the eyeball than that behind it in terms of the anatomic relationship between the eyeball and the orbital bone wall; therefore, the anterior and posterior diameters of the eyeball change significantly more than the vertical and horizontal diameters. The reasons for this might be that the osseous structure of the orbit limits the expansion direction of the eyeball, and some ocular factors might cause different changes in the diameters of highly myopic eyeballs. Assuming that defocusing leads to myopia and that the ocular development may be affected by retinal development, the reason for greater anterior and posterior diameters than vertical and horizontal diameters in highly myopic eyeballs may be that the equator has milder defocusing than the posterior pole [24]. The uneven distribution of retinal growth factors might also affect ocular development [25, 26], but it still lacks strong evidence to support it. Cheng et al. [27] measured the thickness of scleral and choroidal tissues of eight subjects with mild-to-moderate hyperopia, six with emmetropia, and seven with mild-to-moderate myopia, and found that subjects with myopia had thinner sclera and choroid than the subjects of the other two groups, and such extension of sclera and choroid caused an expanded vitreous body.

This study shows that the ocular volume is correlated with diopter, total curvature, and AL. Linear regression equations were also determined. This is supported by Nagra et al. [28], who revealed that the ocular volume could be estimated using the AL. The determination of the ocular volume of a patient with high myopia will enable more efficient planning before surgery, such as customizing an artificial vitreous body that fits the size of the patient’s eyeball and determining the needed amount of silicon oil or expansive gas during surgery. This would be beneficial to the patient’s postoperative recovery and might reduce postoperative complications (e.g., anterior segment ischemia syndrome, postoperative ocular hypertension, postoperative ocular hypotension, and retinal re-detachment). Future studies will have to examine the applicability of those equations in real-world clinical practice. Validation in large cohorts is necessary.

The estimation of the eye volume by CT has been shown to be feasible [29], but it is not practical and should not be done in all patients. Indeed, CT examination and 3D reconstruction require time and human resources, and consuming such resources might be unnecessary if simple and reliable equations can be designed. In addition, radiation exposure remains an issue, particularly in children.

This is an exploratory study, but as high-end CT technology has yet to be widely used in ophthalmic examinations, this study was affected by a small sample size and selection bias. Future studies should include more high myopia patients, including adult patients, to verify and improve the ocular volume estimation model, and promote the application of high-end CT in ophthalmology in selected cases. Currently, studies on ocular morphology require the manual collection of images, which is time and labor cost consuming. We expect big data collection and analysis by artificial intelligence for a way to determine the ocular volume by refractive factors and hope to design patient-friendly surgery for severe eye disease before surgery.

## Conclusions

In children, the ocular volume is negatively and linearly correlated with the diopter and curvature, and positively and linearly correlated with the AL. This will lay a theoretical foundation for future studies on the ocular volume of patients of different diopters.

## Data Availability

The datasets used and/or analyzed during the current study are available from the corresponding author on reasonable request.

## Abbreviations

IOP: Intraocular pressure.
CT: Computed tomography.
